# Bioactivity-guided separation of potential α-glycosidase inhibitor from clerodendranthus spicatus based on HSCCC coupled with molecular docking

**DOI:** 10.1038/s41598-021-86379-9

**Published:** 2021-03-25

**Authors:** Chunsheng Zhu, Hongjuan Niu, Anzheng Nie, Meng Bian

**Affiliations:** 1 The First Affiliated Hospital of Zhengzhou University, Zhengzhou, 450052 China; 2 School of Pharmacy in Minzu University of China, Beijing, 100081 China

**Keywords:** Computational biology and bioinformatics, Drug discovery

## Abstract

*Clerodendranthus Spicatus* is a traditional Dais medi-edible plant and it has been proven to have good blood glucose-lowering efficacy. However, the material basis of *Clerodendranthus Spicatus* has not been clarified yet and therefore needs to be determined. In this paper, the effective ingredients of this medicine were purified by high-speed counter-current chromatography. Alongside, their potential hypoglycemic activity was determined by α-glucosidase inhibitory activities in vitro and molecular docking. Finally, five compounds were purified and identified as 2-caffeoyl-L-tartaric acid (1), N-(E)-caffeoyldopamine (2), rosmarinc acid (3), methyl rosmarinate (4), 6,7,8,3′,4′-Pentamethoxyflavone (5). Examination of α-glucosidase inhibitory activity in vitro showed that 2-caffeoyl-L-tartaric acid and rosmarinic acid had a higher inhibitory activity than acarbose. Molecular docking indicated that the affinity energy of the identified compounds ranged from − 7.6 to − 8.6 kcal/mol, a more desirable result than acarbose (− 6.6 kcal/mol). Particularly, rosmarinc acid with the lowest affinity energy of − 8.6 kcal/mol was wrapped with 6 hydrogen bonds. Overall, α-glucosidase inhibitory activities and molecular docking suggested that rosmarinc acid was likely to be a promising hypoglycemic drug.

## Introduction

Diabetes is a serious chronic metabolic disorder affecting 463 million of individuals worldwide in 2019, and there may be an extra 700 million by 2045^1^. Additionally, numerous epidemiological studies have shown that diabetes is not only closely related to blindness, limb amputations, and renal failure, but also an independent risk factor for peripheral artery disease^2–4^. Diabetes can be categorized into two types: T1D is mainly caused by insufficient insulin secretion, and T2D is abnormal insulin secretion and/or non-insulin-dependent diabetes, complicated by postprandial hyperglycemia^5, 6^. Remarkably, according to clinical statistics, only 5–10% of diabetic patients are affected by T1D, while T2D accounts for more than 90% of diabetes cases^7, 8^.

It is well known that the inhibitors of carbohydrate-related enzymes like α-glycosidase by delaying glucose absorption, including acarbose, miglitol, and voglibose, is one of the most effective controlling methods to overcome postprandial hyperglycemia^8^. However, long-term usage of these drugs exhibits a series of undesired side effects on the digestive system, such as abdominal pain, diarrhoea and nausea^9, 10^. Accordingly, screening new α-glycosidase inhibitors to develop the new drugs with fewer side effects has been a research hotspot in recent years.

*Clerodendranthus spicatus (Thunb.)* C. Y. Wu (*C. spicatus*), popularly known as “orthosiphon” or “kidney tea”, is a perennial herb of the Labiatae family, which is an endemic specie distributed in southern China, such as Yunnan, Fujian and Guangxi provinces^11, 12^. *C. spicatus* is a kind of medi-edible plant medicine, which has been used as an herbal remedy for gout, acute and chronic nephritis in Dai medicine with a history of more than 2000 years in local ethnic groups in Yunnan Province, China^13, 14^. In addition, an earlier report has indicated that this plant contains high amount of flavonoids, phenolic acids, and anthraquinones^15^. Moreover, modern pharmacological studies have shown that the extracts of *C. spicatus* exhibit a significant effect on hypoglycemic effect, which can significantly lower the blood glucose levels in the streptozotocin-induced diabetes mouse model^16^. Therefore, there is a high possibility to screen and isolate potential target therapeutic components from *C. spicatus* against for diabetes.

However, of note, the pharmacological activities were conducted on the complex extracts and it remained unclear which of the components in the crude extracts were active compounds and how each of these active components contributed to the pharmacological effects. As such, in order to well study the pharmacological activities, large amount of these compounds is needed, and setting up an efficient method for separation of the compounds is a major task. As a chromatography that requires no solid packing, high-speed counter-current chromatography (HSCCC) is not limited by the solid stationary phase and has a larger sample load, and the use of solvent system avoids the use of solid chromatographic fillers and reduces the separation cost^17^. Meanwhile, the selection of thousands of different proportional solvent systems increases the possibility of successful separation of compounds with polar similar by HSCCC^18, 19^. Thus, HSCCC gradually became a crucial technique for separating the compounds from the natural products^20^.

Nowadays, the studies of active compounds and efficacy-organ/tissue-cell-receptor/channel are faced with the adversity of high cost^21^. Fortunately, the interaction of the screened compounds and the potential targets can be predicted by molecular docking. Docking analyses were carried out for all molecules included in this study to predict the interaction between ligand and active site of receptor by energy-based scoring function^22^. Therefore, in the present study, we separated and purified the target compounds of *C. spicatus* by HSCCC, and then evaluated the interaction between α-glycosidase and the target compounds by α-glucosidase inhibitory activities and molecular docking, so as to provide reference for the development of new diabetes drugs.

## Results and discussion

### Selection of solvent system

A successful separation of target compounds using HSCCC requires a careful search for a suitable two-phase solvent system that could provide an ideal range of partition coefficients (K). K values in the range of 0.2 to 5.0 are generally considered to be appropriate for HSCCC separation^20^. In addition, the separation factor between the two components (α = K1/K2, K1 > K2) should be greater than 1.5^23^. Furthermore, the target compounds must be stable and soluble in such a solvent system, and the solvent system must be separated clearly and quickly into upper and lower phase in 30 s^20^.

More recently, Liang et al.^24^ successfully developed a new solvent selection strategy for targeted counter-current chromatography purification of natural products based on HEMWat 9 × 9 map. Particularly, it is easier to get a suitable solvent system through making a simple screening of 2 ~ 4 HEMWat two-phase solvent systems to obtain the sweet line or sweet zone without special knowledge, which is of great help to the separation of traditional Chinese medicine. Hence, in the present paper, an HEMWat 9 × 9 map-based solvent selection strategy was used. The key of the solvent selection strategy is to establish a linear regression equation using two solvent systems in the same line-group. Based on the diagonal lines of the HEMWat 9 × 9 map, HEMWat (1:5:1:5) (I) and HEMWat (5:5:5:5, v/v) (II) were chosen to find the linear regression equation of log *K* versus content of hexane number of solvent systems. According to the K value of the compounds, we find that the K value of compound 1 is minimum in the HEMWat. Thus, the compound 1 was selected as standard compound for linear regression. The K values of the standard compound were measured respectively with KI at 0.71 and KII at 2.04 respectively. Then, a linear regression equation “y = 0.1549x − 0.4647” was obtained, where x is the hexane number of the solvent system and y is the log K. According to the regression equation, the appropriate system (K = 1) was determined as HEMWat (3:5:3:5, v/v). Further, the *K* value of the standard compound 1 in the solvent system was determined to be 1.13 which was very close to the predicted value. Thus, the solvent system was confirmed for further study.

Then, K values of the target compounds were confirmed. As shown in Table 1, the α values between compounds 3 and 2 was lower than 1.2, which indicated that the baseline separation of two compounds cannot be achieved in one step. Therefore, an appropriate polarity regulator was needed to alter the distribution of the target compounds. In the study, the acetic acid was used to change the distribution of the target compounds in the solvent system. Different volume ratios of acetic acid were added in solvent system HEMWat (3:5:3:5, v/v). As shown in Table 1, with the increasing of the acetic acid, the K value of compound 1 almost keep unchanged, while the K value of compounds 2, 3 and 4 were decreased clearly. In the solvent system HEMWat (3:5:3:5, v/v), K_4_ was higher than K_2,_ with the acetic acid added in, the K_2_ was higher than K_4_, which indicated that the distribution of compounds 2 and 4 in the solvent system were reversed. As shown in Table 1, the K value of the target compounds were all in a proper range, but the α of compounds 2 and 4 in HEMWat (3:5:3:5, v/v) (0.5% acetic acid), and the α of compounds 2 and 3 in n-hexaneHEMWat (3:5:3:5, v/v)(1.0% acetic acid) were both lower than 1.2, which indicated that it was difficult for these compounds to achieve baseline separation during the HSCCC. Fortunately, the target compounds had proper K values and α values in HEMWat (3:5:3:5, v/v) (1.5% acetic acid). Thus, acetic acid can be used as an effective solvent system polarity regulator and HEMWat (3:5:3:5, v/v) (1.5% acetic acid) was finally chosen to be the final choice for HSCCC process.

**Table 1:** The *K* values of the target compounds.

Solvent system		*K*_*1*_	*K*_*2*_	*K*_*3*_	*K*_*4*_
HEMWat	3:5:3:5	1.13	4.94	2.64	5.23
HEMWat (0.5% acetic acid)	3:5:3:5	1.13	3.57	2.59	3.48
HEMWat (1.0% acetic acid)	3:5:3:5	1.09	2.33	2.41	2.11
HEMWat (1.5% acetic acid)	3:5:3:5	0.98	1.93	2.34	1.48

### HSCCC process

**Figure 1:**
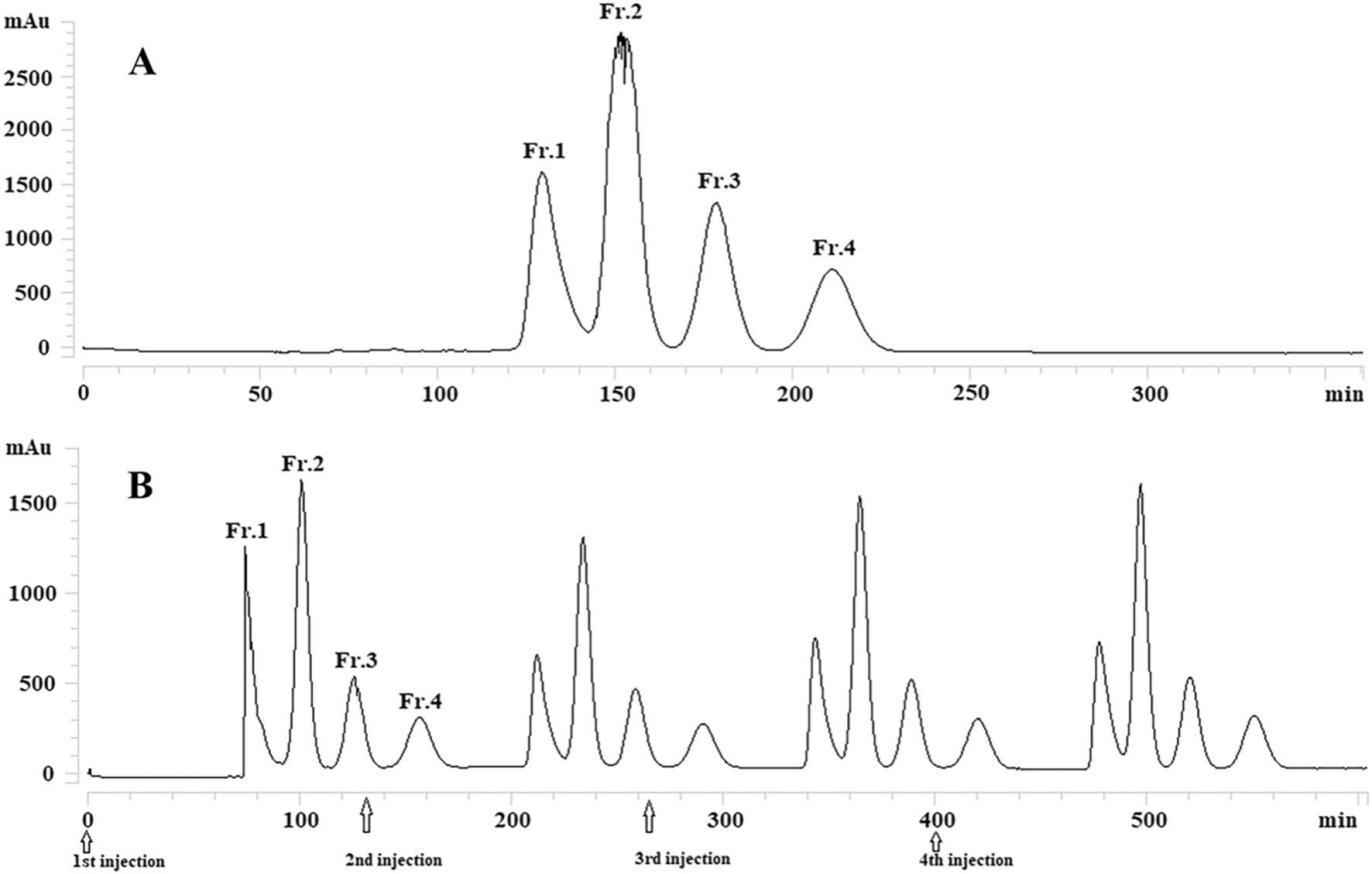
HSCCC chromatogram of 40% methanol fraction using n-hexane–ethyl acetate–methanol–water (3:5:3:5, v/v) (1.5% acetic acid). Conditions: stationary phase, upper phase; revolution speed, 800 rpm; separation temperature, 30 °C; detection wavelength, 320 nm; flow rate, 2.0 ml/min (**A**: Single injection; **B**: forth continuous sample injection).

**Figure 2:**
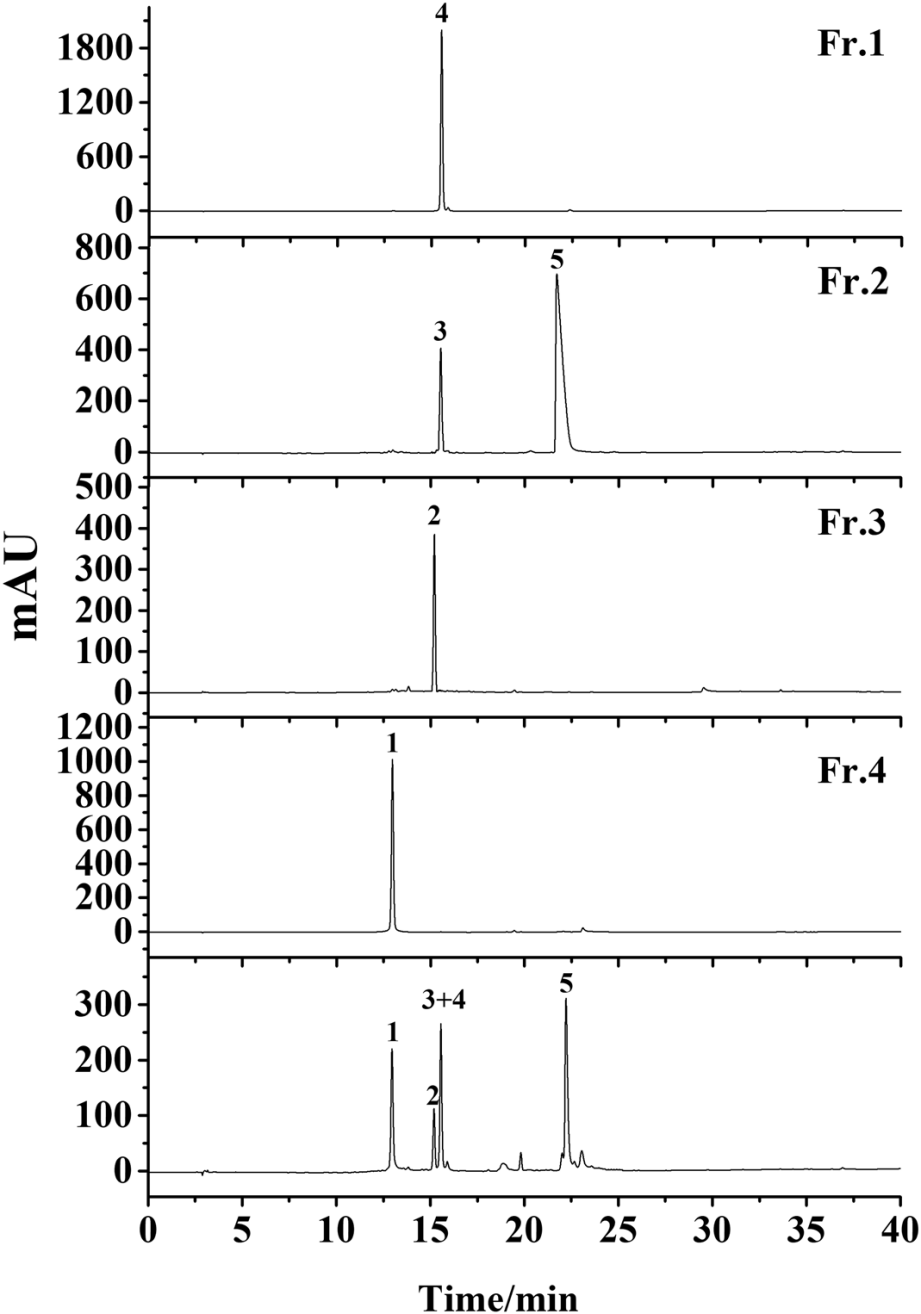
HPLC analysis of four fractions from HSCCC. Conditions: column, Platisil ODS-C18 analytical column (250 mm × 4.6 mm i.d., 5 μm); mobile phase, 0.1% acid water (**A**) and methanol (**B**), gradient elution program: 0–30 min, 10–95% B; 30–40 min, 95% B; flow rate, 1.0 mL/min; temperature, 30 °C; detection wavelength, 254 nm.

The HSCCC process was carried out using HEMW at (3:5:3:5, v/v) (1.5% acetic acid) with other parameters: revolution speed, 800 rpm; flow rate, 2.0 mL/min; temperature, 30 °C; 20 mg of the sample was dissolved in 10 mL of the lower phase with forth continuous sample injection in a single run (Fig. 1). There are four fractions collected and analyzed by HPLC (Fig. 2). Under the condition, 26 mg of compound 1, 42 mg of compound 3 + 5, 17 mg of compound 2, and 23 mg of compound 4 was obtained from 160 mg of sample. HPLC analysis showed that the purities of compounds 1, 2, 4 were higher than 98%. And the fraction of compounds 3 + 5 was further purified by preparative HPLC (prep-HPLC).

### prep-HPLC purification

**Figure 3:**
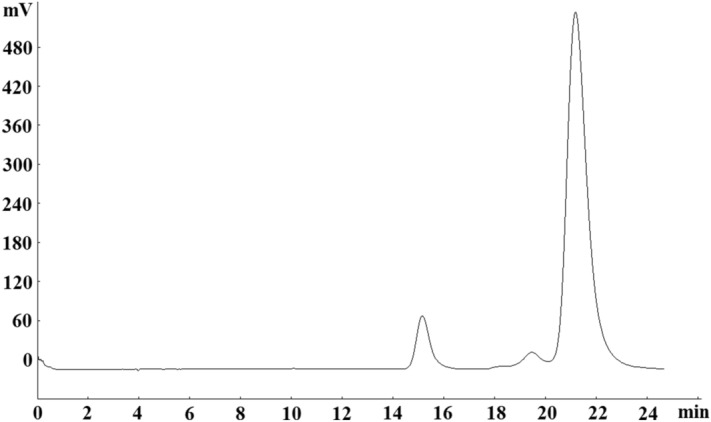
Prep-HPLC chromatogram of fraction 2 from HSCCC. Conditions: column, Reprosil 100 C18 column (250 × 20 mm i.d., 10 μm); mobile phase, 40% methanol; flow rate, 18 mL/min; detection wavelength, 254 nm.

**Figure 4:**
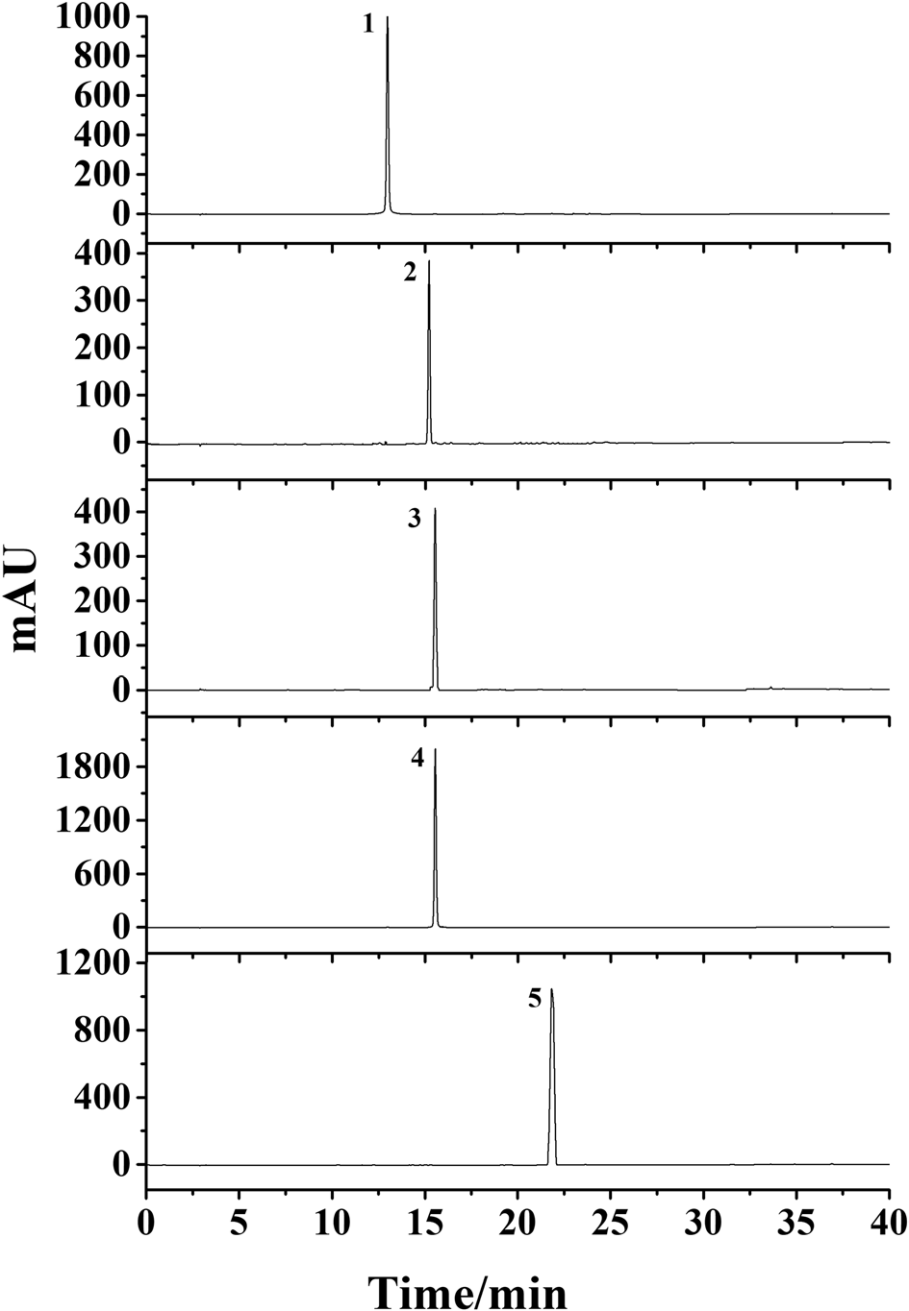
Purity detection of the five compounds by HPLC. Conditions: column, Platisil ODS-C18 analytical column (250 mm × 4.6 mm i.d., 5 μm); mobile phase, 0.1% acid water (**A**) and methanol (**B**), gradient elution program: 0–30 min, 10–95% B; 30–40 min, 95% B; flow rate, 1.0 mL/min; temperature, 30 °C; detection wavelength, 254 nm.

Because prep-HPLC has excellent column efficiency, high-throughput purification, and separation reproducibility, it is widely used in the separation of herbal medicines^25^. Hence, in the present study, prep-HPLC was used for further purification of these two compounds (compounds 3 + 5). From the condition of HPLC analysis of the sample, target compounds could be eluted when the concentration of methanol reached 40%. Thus, isocratic elution modes with 40% methanol were adopted in our experiment (Fig. 3). As a result, the target compounds were well separated, and 8 mg compound 3 and 40 mg compound 5 were obtained. HPLC analysis (Fig. 4) showed that the purities were higher than 98% after prep-HPLC purification.

### Structural identification

**Figure 5:**
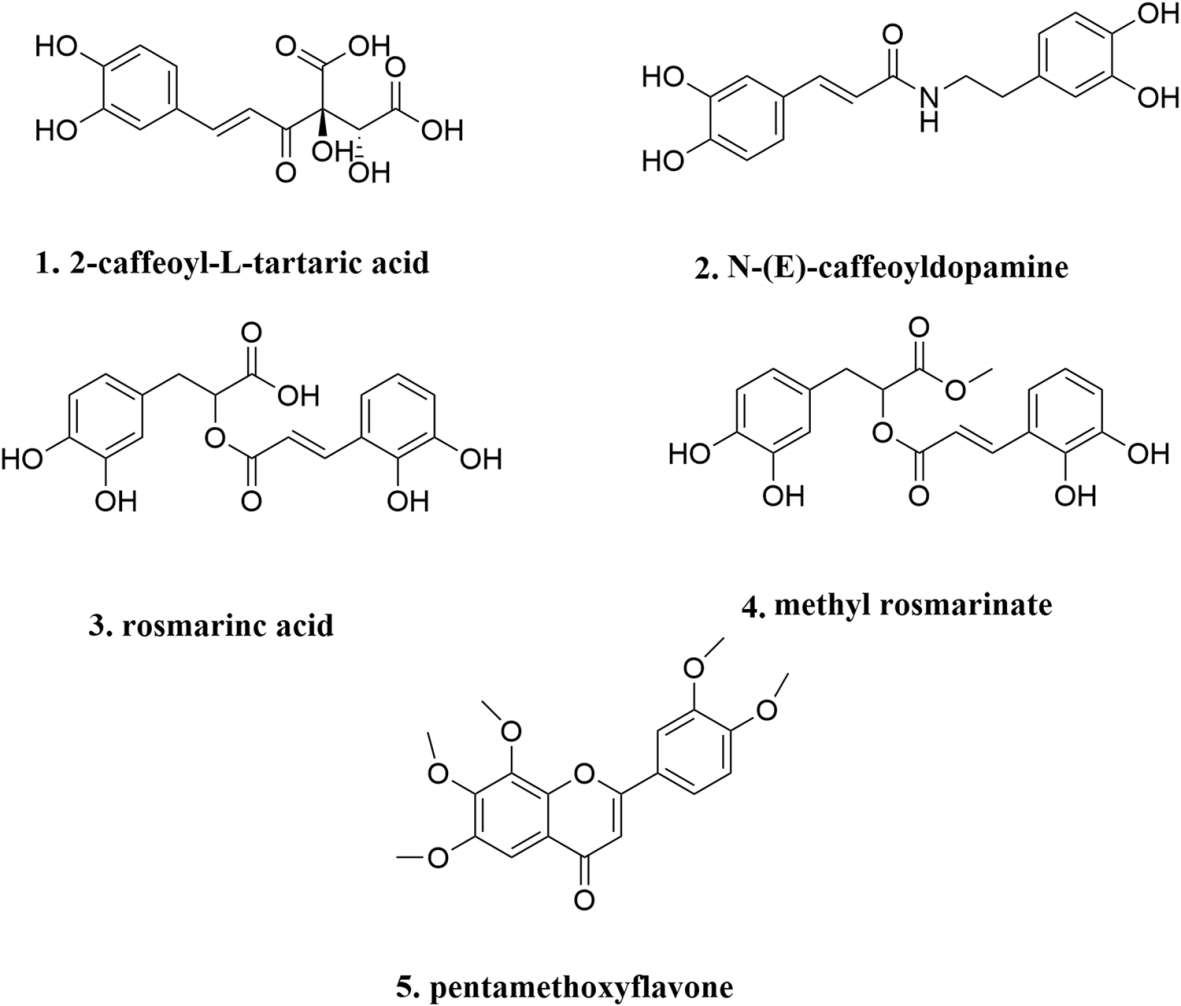
The chemical structures of the five compounds.

The chemical structures of the five compounds were elucidated by ^1^H-NMR and ^13^C-NMR and were identified as 2-caffeoyl-L-tartaric acid (1), N-(E)-caffeoyldopamine (2), rosmarinc acid (3), methyl rosmarinate (4), 6,7,8,3′,4′-pentamethoxyflavone (5) (Fig. 5). Compounds 3 and 5 were separated from *C. spicatus* for the first time. The detailed data were as follows:

2-Caffeoyl-L-tartaric acid (1): 1H NMR (400 MHz, DMSO-*d*_6_): δ 12.97 (1H, s, 5-OH), 10.42 (1H, s, 4′-OH), 7.44 (2H, d, J = 8.1 Hz, H-2′, 6′), 6.88 (2H, d, J = 8.1 Hz, H-5′, 3′), 6.71 (1H, s, H-3), 6.70 (1H, d, J = 2.0 Hz, H-8), 6.42 (1H, d, J = 2.1 Hz, H-6), 5.04 (1H, s, H-1″). 13C NMR (100 MHz, DMSO-*d*_6_): δ 182.58 (C-4), 164.37 (C-2), 163.01 (C-7), 161.46 (C-5), 161.13 (C-4′), 158.90 (C-9), 128.82 (C-2′, 6′), 119.62 (C-1′), 116.38 (C-3′, 5′), 113.61 (C-10), 102.49 (C-3), 99.67 (C-1″), 99.41 (C-6), 91.93 (C-8), 77.75 (C-5″), 77.02 (C-3″), 74.61 (C-2″), 70.38 (C-4″), 61.34 (C-6″). Compared with the results in the literature^26^, it was identified as 2-caffeoyl-L-tartaric acid.

N-(E)-caffeoyldopamine (2): ^1^H NMR (400 MHz, DMSO-*d*_6_) δ: 3.06–3.43 (6H, m, glc-1″, 2″, 3″, 4″, 5″, 6″), 3.91 (3H, s, H-7-OMe), 5.04 (1H, d, J = 7.2 Hz, H-1″), 6.74 (1H, s, H-3), 6.88 (1H, s, H-8), 6.91 (1H, d, J = 8.2 Hz, H-3′), 7.46 (2H, m, H-2′, 6′), 9.43 (1H, s, H-3′-OH), 10.01 (1H, s, H-4′-OH), 13.09 (1H, s, H-5-OH). ^13^C NMR (100 MHz, DMSO-*d*_6_) δ: 56.54 (C-OMe), 60.88 (C-6″), 69.75 (C-2″), 74.14 (C-4″), 76.55 (C-5″), 77.29 (C-3″), 91.52 (C-8), 101.99 (C-1″), 102.70 (C-3), 104.39 (C-10), 113.52 (C-2′), 115.94 (C-5′), 119.05 (C-6′), 121.47 (C-1′), 128.10 (C-6), 145.77 (C-3′), 149.80 (C-4′), 151.70 (C-9), 152.57 (C-5), 158.51 (C-7), 164.19 (C-2), 182.13 (C-4). Compared with the results in the literature^27^, it was identified as N-(E)-caffeoyldopamine.

Rosmarinc acid (3): ^1^H NMR (400 MHz, DMSO-*d*_6_) δ: 7.56 (1H, d, J = 2.1 Hz, H-2′), 7.53 (1H, dd, J = 2.1, 8.1 Hz, H-6′), 6.84 (1H, d, J = 8.2 Hz, H-5′), 6.39 (1H, d, J = 1.9 Hz, H-8), 6.19 (1H, d, J = 1.9 Hz, H-6), 5.33 (1H, d, J = 7.2 Hz, H-1″), 4.39 (1H, s, H-1″), 3.71 (2H, d, J = 9.7 Hz, H-6″), 1.00 (3H, d, J = 6.1 Hz, CH_3_-6‴). ^13^C NMR (100 MHz, DMSO-*d*_6_) δ: 177.78 (C-4), 164.67 (C-7), 161.65 (C-5), 157.02 (C-9), 156.87 (C-2), 148.89 (C-4′), 145.21 (C-3′), 133.73 (c-3), 122.04 (C-6′), 121.60 (C-1′), 116.69 (C-5′), 115.67 (C-2′), 104.35 (C-10), 101.65 (C-1″), 101.20 (C-1‴), 99.36 (C-6), 94.06 (C-8), 76.89 (C-3″), 76.33 (C-5″), 72.29 (C-2″), 71.00 (C-4‴), 70.82 (C-3‴), 70.45(C-2‴, C-5‴), 68.70 (C-4″, C-6″), 18.20 (C-6‴). Compared with the results in the literature^28^, it was identified as rosmarinc acid.

Methyl rosmarinate (4): ^1^H NMR (400 MHz, DMSO-*d*_6_) δ: 1.83 (3H, s, H-2‴), 3.11 ~ 3.42 (6H, m, glc-1″, 2″, 3″, 4″, 5″, 6″), 3.90 (3H, s, H-7-OMe), 4.95 (1H, d, J = 7.2 Hz, H-1″), 6.72 (1H, s, H-3), 6.85(1H, s, H-8), 6.88 (1H, d, J = 8.2 Hz, H-3′), 7.43 (2H, m, H-2″,6′), 9.39 (1H, s, H-3′-OH), 10.00 (1H, s, H-4′-OH), 13.05 (1H, s, H-5-OH). ^13^C NMR (100 MHz, DMSO- *d*_6_) δ: 20.44 (C-2‴), 56.46 (C-OMe), 63.21 (C-6″), 70.03 (C-2″), 74.14 (C-2″), 76.55 (C-5″), 77.29 (C-3″), 91.52 (C-8), 102.09 (C-1″), 102.68 (C-3), 104.39 (C-10), 113.32 (C-2′), 115.85 (C-5′), 118.94 (C-6′), 121.28 (C-1′), 127.75 (C-6), 145.48 (C-3′), 149.74 (C-4′), 151.61 (C-9), 152.61 (C-5), 158.43 (C-7), 163.93 (C-2), 169.91 (C-1‴), 181.13 (C-4). Compared with the results in the literature^29^, it was identified as methyl rosmarinate.

6,7,8,3′,4′-Pentamethoxyflavone (5): ^1^H NMR (400 MHz, DMSO-*d*_*6*_) δ: 13.08 (OH-5), 10.36 (OH-4′), 7.98 (2H, d, J = 8.8 Hz, H-2′, H-6′), 6.94 (1H, s, H-8), 6.93 (2H, d, J = 8.8 Hz, H-3′, H-5′), 6.86 (1H, s, H-3), 5.14 (1H, m, OH-2″), 5.06 (1H, m, OH-3″), 5.04 (1H, m, OH-4″), 4.98 (1H, d, J = 5.2 Hz, OH-1″), 4.30 (1H, m, OH-5″), 4.13 (1H, dd, J = 5.0, 10.0 Hz, OH-6a''), 3.91 (3H, s, OCH_3_-7), 3.59 (1H, dd, J = 4.5, 11.0 Hz, OH-6b''), ^13^C NMR (100 MHz, DMSO-*d*_*6*_) δ:182.71 (C-4), 164.48 (C-2), 161.76 (C-4′), 159.00 (C-7), 153.07 (C-9), 152.15 (C-5), 129.00 (C-2′, C-6′), 128.58 (C-6), 121.58 (C-1), 116.43 (C-3′, C-5′), 105.37 (C-10), 103.16 (C-1″), 102.46 (C-3), 92.12 (C-8), 77.76 (C-3″), 77.02 (C-5″), 74.61 (C-2″), 70.30 (C-4″), 61.34 (C-6″), 57.03 (OCH_3_-7). Compared with the results in the literature^30^, it was identified as 6,7,8,3′,4′-pentamethoxyflavone.

### α-Glycosidase inhibitory activities

The α-glycosidase enzyme hydrolyzes polysaccharides and oligosaccharides into monosaccharides units, which plays a key role in delivering the glucose into the blood^31^. Consequently, it is recommended that one of the treatments of T2D is to delay the digestion of carbohydrates by inhibiting α-glycosidase enzyme, thereby reducing postprandial hyperglycemia^32^. In this study, the α-glycosidase inhibitory activity of five compounds was examined and the results were shown in Table 2. This evaluation showed that none of the N-(E)-caffeoyldopamine, methyl rosmarinate, and 6,7,8,3′,4′-Pentamethoxyflavone had a significant activity. However, the most effective α-glycosidase inhibitory values were obtained by 2-caffeoyl-L-tartaric acid and rosmarinic acid, with inhibitory values of 69.85 ± 1.27% and 71.06 ± 1.82%, respectively, both higher than that of acarbose.

**Table 2:** α-Glycosidase inhibitory activity of five compounds.

Compounds	α-Glycosidase inhibitory (%)
Acarbose	66.21 ± 2.91
2-Caffeoyl-L-tartaric acid	69.85 ± 1.27
N-(E)-caffeoyldopamine	53.73 ± 5.81
Rosmarinc acid	71.06 ± 1.82
Methyl rosmarinate	39.66 ± 4.49
6,7,8,3′,4′-Pentamethoxyflavone	23.07 ± 0.61

Notably, numerous studies have extensively been conducted to investigate the pharmacological activities of rosmarinic acid, particularly for anti-diabetic property. Previous studies reported that rosmarinic acid could exhibit significant inhibition of α-glucosidase^33–35^. Furthermore, rosmarinic acid was also found to reduce hyperglycemia and boost insulin sensitivity by activating glucose transporter type 4^26^. Interestingly, a recent study investigated by Ruiz-Vargas et al. showed that rosmarinic acid caused a greater decrease in blood glucose, when compared to that of acarbose. Concurrently, rosmarinic acid did not show a greater decrease in blood glucose at 5 mg/kg than 0.5 mg/kg, indicating that rosmarinic acid is not dose-dependent^34^. Collectively, these results suggest that rosmarinic acid is a promising agent for treating T2D. Nevertheless, documentations of the role of 2-caffeoyl-L-tartaric acid in the anti-diabetic property are lacking, and further studies are still needed.

### Molecular docking result

Mostly, molecular docking studies are applied to drug development, including the discovery of novel α-glycosidase inhibitors. Furthermore, molecular docking also predicts the binding orientation of the ligands to the active sites of α-glycosidase frequently^36^. In this process, AutoDock Vina is proved to be a powerful tool for evaluating the binding efficacy between ligands and protein targets^37^. Therefore, in the present study, AutoDock Vina v1.1.3 was applied to simulate the molecular recognition process between α-glycosidase and the compounds separated above from *C. spicatus* and the binding energies were calculated simultaneously.

**Figure 6:**
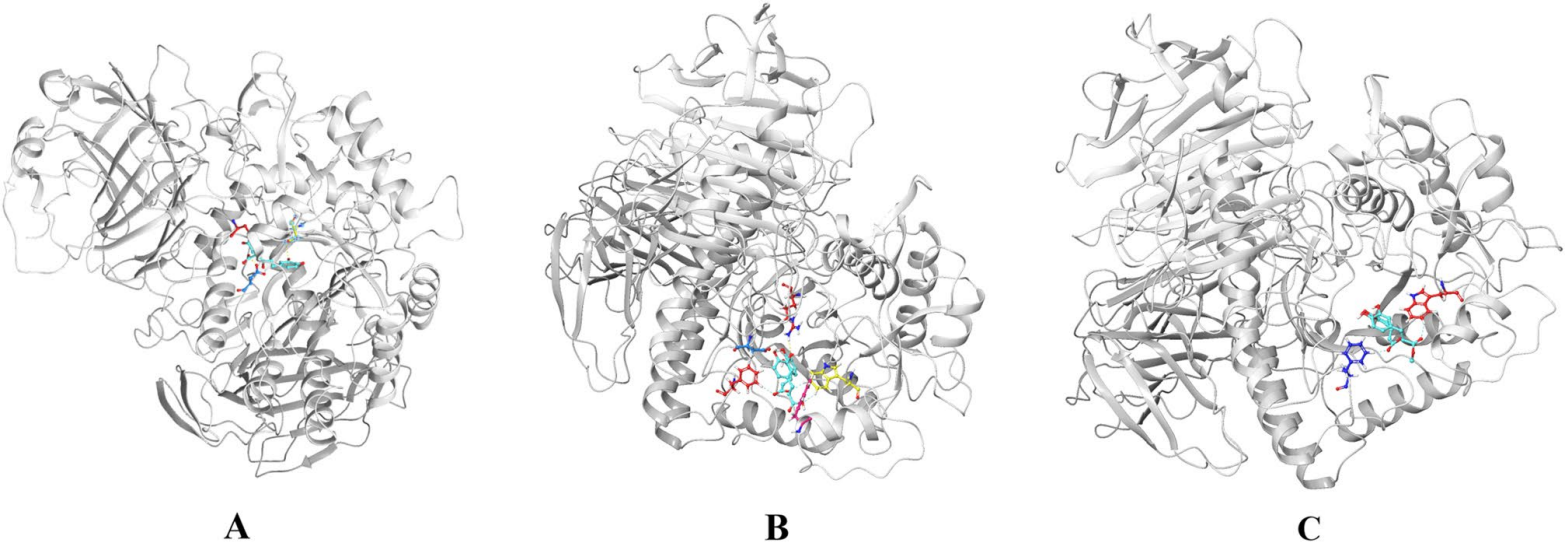
Molecular docking models of α-glycosidase and compounds 1 (**A**), 3 (**B**), and 4 (**C**).

Molecular docking results details are listed in Table 3. The affinity energy of the identified compounds ranges from − 7.6 to − 8.6 kcal/mol, a more desirable result than acarbose (− 6.6 kcal/mol), indicating that those five compounds may have a potential to be a α-glycosidase inhibitor. In addition, those compounds can be embedded in the α-glycosidase cavity, and the compound 1, 3, and 4 are predominantly stable in the catalytic site by hydrogen bond as shown in Fig. 6. Figure 6A exhibits the interactions of 2-caffeoyl-L-tartaric acid with α-glycosidase residues through hydrogen bonds with ASP1526, ASP1420 and ASP1157, showing a binding energy of − 7.7 kcal/mol. Similarity, methyl rosmarinate (Fig. 6C) displays a binding energy of − 8.4 kcal/mol, presenting hydrogen bonds with residues TRP1355 and PHE1560. In parallel, compared with other compounds, compounds 3 (− 8.6 kcal/mol) and 4 (− 8.4 kcal/mol) show lower affinity energy. Based on the phenomenon, one of the reasons may be that they form hydrogen bonds with α-glycosidase through PHE1560 and TRP1355, respectively.

**Table 3:** The docking results of the five compounds to α-glycosidase.

Compounds	Affinity kcal/mol	Number of H-bonds	Bonding residues	Bond length
2-Caffeoyl-L-tartaric acid	− 7.7	3	ASP 1526 ASP 1420 ASP 1157	2.27 2.19 2.71
N-(E)-caffeoyldopamine	− 8.3	–	–	–
Rosmarinc acid	− 8.6	6	ARG 1510 ASP 1526 PHE 1560 TRP 1355 TRP 1369	2.77 1.86 2.25 2.48, 2.49 2.04
Methyl rosmarinate	− 8.4	2	TRP 1355 PHE 1560	2.41
				2.14
6,7,8,3′,4′-Pentamethoxyflavone	− 7.6	–	–	–

“–” means no.

It is also noteworthy that, in contrast to the inhibitory activity of α-glycosidase in vitro, the binding energy of compound 2 towards α-glycosidase is too low (− 8.5 kcal/mol), indicating strong binding affinity for α-glycosidase. This is not surprising, because the results of molecular docking are not always consistent with experiments in vitro^38^. Additionally, the lack of hydrogen bond interaction between compound 2, 4 and α-glycosidase show that the compound 2–α-glycosidase and compound 4–α-glycosidase complexes might be stabilized by other non-covalent forces such as hydrophobic interactions.

**Figure 7:**
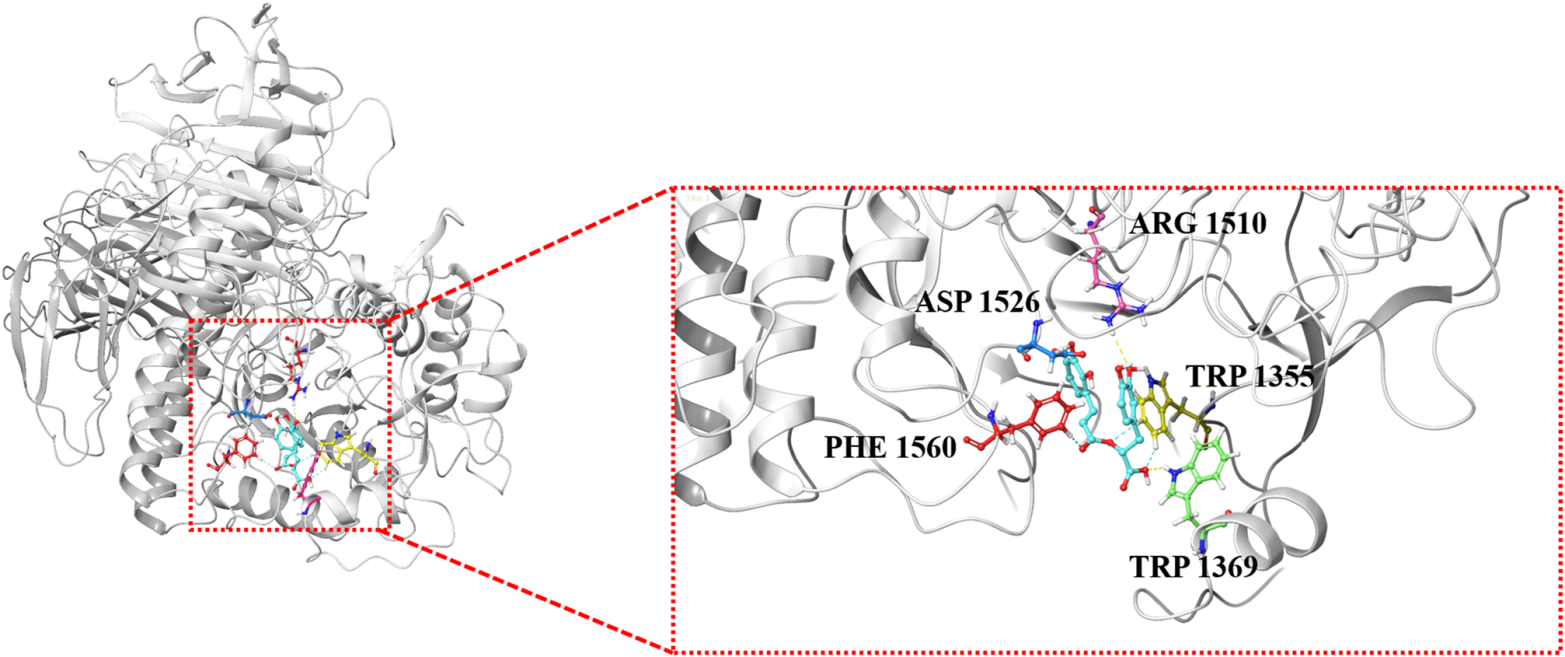
Molecular docking models of α-glycosidase and compound 3 with overall and enlarged view.

Particularly, rosmarinc acid (compound 3) with the affinity energy of − 8.6 kcal/mol occupied the active region by interfering with amino acid residues located in the binding cavity, including ARG 1510, ASP 1526, PHE 1560, TRP 1355, and TRP 1369 (Fig. 6B). In the conformation of α-glycosidase-compound 3 complex, there are 6 hydrogen bonds. As shown in Fig. 7, two hydroxyl groups of the compound 3 formed the hydrogen bonds with the side-chain amino-group of ARG 1510 and TRP 1369, and the length of hydrogen bonds were 2.77 Å and 2.04 Å respectively. Simultaneously, another hydroxyl group of the compound 3 formed the hydrogen bond with the side-chain carboxyl group of ASP 1526, and the length of hydrogen bond was 1.86 Å. Besides, the 3 hydrogen bonds were formed by side-chain aromatic hydrogen of PHE 1560 and TRP 1355 with the oxygen of the ligand molecule, and the bond length were 2.25 Å, 2.49 Å and 2.48 Å, respectively (Fig. 7). These hydrogen bonds overtly strengthened the interaction and served as anchors for binding the inhibitor in the active site. It is important to note that the interaction with ASP 1526, PHE 1560 and TRP 1369 has been described as characteristic of α-glycosidase partial inhibitor^39^. Moreover, this finding indirectly verified that rosmarinc acid had a regulatory effect on α-glycosidase. Therefore, *C. spicatus* may be a good source for isolating rosmarinc acid, and for the development of new hypoglycemic drugs.

## Conclusion

A simple, rapid and efficient method using HSCCC with continuous sample injection was established for separation of five compounds with similar polarity from *C. spicatus*. Five compounds, including 2-caffeoyl-L-tartaric acid, N-(E)-caffeoyldopamine, rosmarinc acid, methyl rosmarinate and 6,7,8,3′,4′-pentamethoxyflavone were isolated from *C. spicatus* through HSCCC. Furthermore, the α-glucosidase inhibitory activities in vitro and result of molecular docking of all isolated compounds were evaluated. The result showed that 2-caffeoyl-L-tartaric acid and rosmarinic acid revealed potential hypoglycemic bioactivity. In short, the current study advances our understanding of the chemical composition and biological effects of *C. spicatus.* Alongside, the above compounds, especially rosmarinic acid, can be used to in-depth study for their inhibition of α-glycosidase and further for dietetic approaches designed to manage T2D.

## Materials and methods

### Plant materials

*C. spicatus* was purchased from the herb market of Bozhou, Anhui, China, in September 2019, and identified by Dr. X.P. Li. The dried whole plant (1.0 kg) of *C. spicatus* was crashed to coarse powder.

### Reagents

Analytical grade reagents were used in extraction, MCI, HSCCC separation, which were purchased from the Jinan Reagent Factory (Jinan, China). Chromatographic grade reagents were used for HPLC analysis, and were purchased from Yuwang Chemical Ltd. (Shandong, China). Deionized water was used throughout the experiment.

### Apparatus

The HSCCC used in the study is TBE-300B system with three polytetrafluoroethylene preparative coils (i.d. of the tubing = 1.6 mm, total volume = 280 mL) and a sample loop (20 mL) equipped with a model TBP5002 constant-flow pump (Tauto BiotechCo. Ltd., Shanghai, China), a model UV-500 detector (XUYUKJ Instruments, Hangzhou, China), and a model N2000 workstation (Zhejiang University, Hangzhou, China). ADC-0506 constant temperature-circulating implement (Shanghai Shunyu Hengping Instruments, Shanghai, China) was used to adjust the experiment temperature. The recycling HSCCC separation was carried out on TBE-300B system with a 6-port valve.

HPLC analysis was performed on an Agilent 1260 system (Agilent Technologies Co. Ltd., USA), which was equipped with a G1311C solvent delivery unit, a G1315D UV-DAD detector, a G1316A column thermostat, a G1329B auto sampler, and an Agilent HPLC workstation.

The nuclear magnetic resonance (NMR) spectrometer used in this study was a Mercury-400B NMR spectrometer (Varian Co. Ltd., USA).

### Preparation of crude sample

The crude sample of *C. spicatus* powder (1.0 kg) was powdered and extracted with 70% ethanol (10 L × 3) ultrasonic extraction for 1 h. All filtrates were combined and concentrated to remove ethanol under reduced pressure by rotary evaporation at 60 °C. The water solution was treated by vacuum freeze dryer at 5 °C to obtain crude extract.

**Figure 8:**
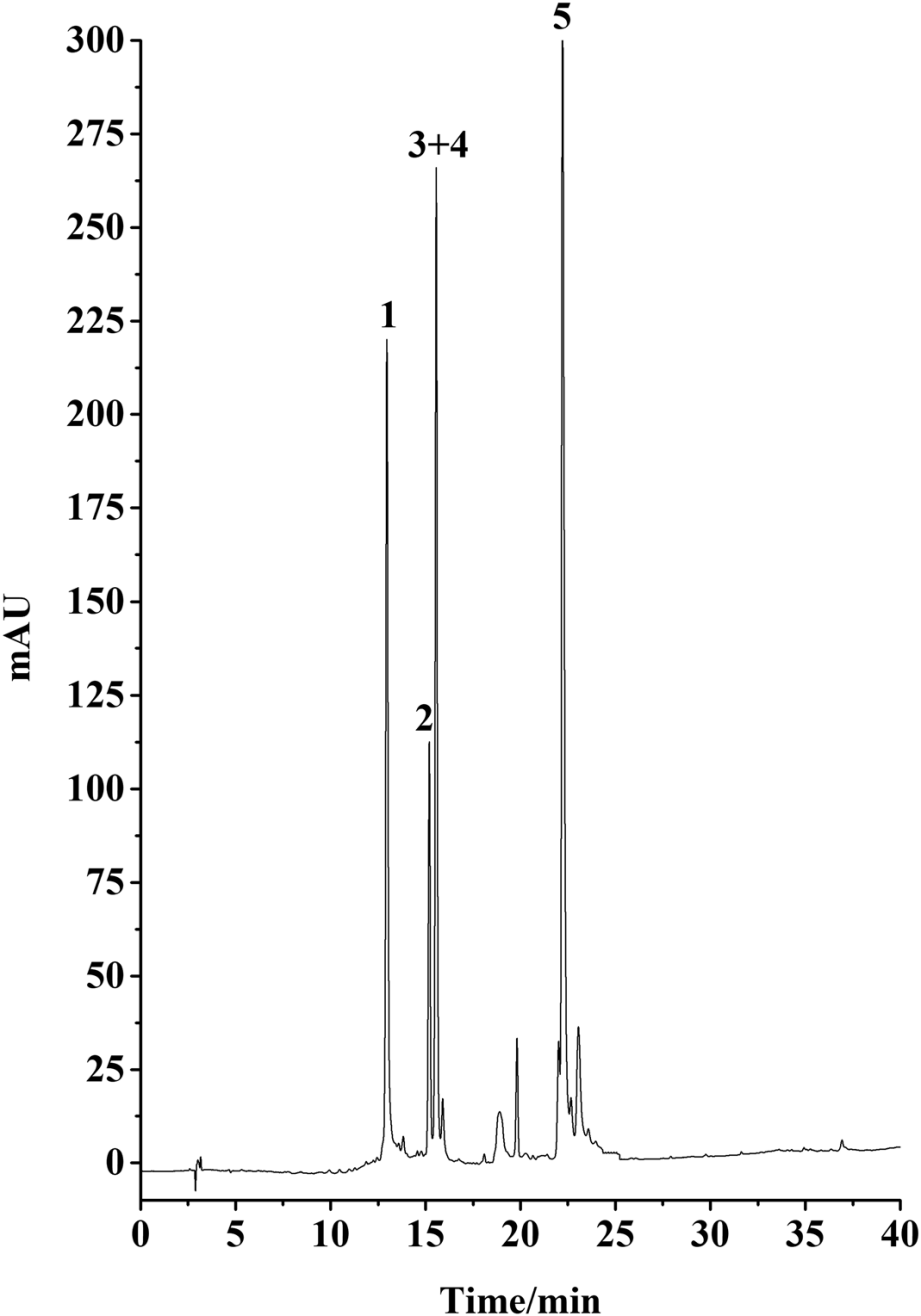
HPLC chromatogram of 40% methanol fraction from MCI column. Conditions: column, Platisil ODS-C18 analytical column (250 mm × 4.6 mm i.d., 5 μm); mobile phase, 0.1% acid water (**A**) and methanol (**B**), gradient elution program: 0–30 min, 10–95% B; 30–40 min, 95% B; flow rate, 1.0 mL/min; temperature, 30 °C; detection wavelength, 254 nm.

10 g of crude sample was dissolved in 50 mL of water, and thenwas injected into the MCI column. Subsequently, the column was eluted by 20%, 40%, 60% and 80% methanol consecutively. The chromatogram was recorded at 254 nm. Each fraction from MCI was analyzed by HPLC. 40% methanol fraction from MCI column (Fr4) was chosen for further experiment (Fig. 8).

### HSCCC process for separation of polarity similar compounds

#### Selection of two-phase solvent system

The solvent system used in HSCCC is based on the partition coefficient (*K*), and was selected by a 9 × 9 map-based solvent selection strategy^23^, and the *K* of target compounds were determined by HPLC method.

#### Preparation of two-phasesolvent system and sample solution

Hexane/ethylacetate/methanol/water (HEMWat) (1.5% acetic acid) (3:5:3:5, v/v) was used in HSCCC process. The solvent system reached equilibrium at room temperature by fully shaken. The upper phase and the lower phase were then separated and degassed via ultrasonic bath for 30 min before use.

In HSCCC process, 20 mg of the sample was dissolved in 10 mL lower phase of the solvent system.

#### HSCCC separation procedure

HSCCC process was performed by the following steps. Firstly, the upper phase used as stationary phase was pumped into the multilayer column. Then, the apparatus was rotated at 800 rpm and the lower phase used as mobile phase was pumped into the column simultaneously. When hydrodynamic equilibrium was reached, sample solution was injected. During separation process, continuous injection system was used for the enrichment of target compounds. The data was detected by a UV-500 detector and peak fractions were collected according to the HSCCC chromatogram.

### HPLC analysis and identification of target compounds

HPLC is used to analyse the 40% ethanol fraction of the MCI resin, fractions of HSCCC and prep-HPLC. A Platisil ODS-C18 analytical column (4.6 × 250 mm, 5 μm) was used. Mobile phase was composed of 0.1% acid water (A) and methanol (B) with a gradient elution program: 0–30 min, 10–95% B; 30–40 min, 95% B. The flow rate was 1.0 mL/min, the column temperature was 30 °C and the detection wave length was 254 nm.

The chemical structures were elucidated by ^1^H-NMR and ^13^C-NMR.

### α-Glycosidase inhibitory activity

The α-Glycosidase inhibitory activity was assessed using a previous method reported by Zhao et al.^40^, with minor modifications. First, 50 μL of the test compound (5 mg/mL) and 50 μL of α-glucosidase (0.5 U/mL) were preincubated in 96-well plate at 37 °C for 10 min. Then, the reaction was initiated by adding 50 μL of 0.5 mmol/mL p-nitrophenyla-D-glucopyranoside as substrate. Subsequently, the 96-well plate was incubated for an additional 20 min at 37 °C, which was followed by the adding 50 μL of 0.1 mol/L Na_2_CO_3_ to stop the reaction. The absorbance values were measured under 405 nm spectrophotometrically according to previous studies^41, 42^. Acarbose was used as positive control. All experiments were repeated five times. The calculation formula of the inhibition rate is as follows: Inhibition rate (%) = (Control absorbance values -Test absorbance values/Control absorbance values) × 100.

### Molecular docking

Molecular docking was chosen for understanding the mode of interactions between target compounds and α-glucosidase. The docking program was performed through using AutoDock Vina v1.1.3. Firstly, the structures of these compounds were saved as a docking ligand in PDB format, and the energy of ligand molecules was minimized by using Chem 3D 16.0. The X-ray crystal structure of α-glycosidase (PDB ID: 3TOP) was download from RSCB database. Secondly, hydrogen atoms were added to the protein structure to ensure the correct protonation states. Then, all ligands including the protein were converted to PDBQT format. Concurrently, the docking analysis was performed in a grid map of 18 × 20 × 20 with the spacing of 1 Å centered on the active site of α-glycosidase. Finally, the docking results were visualized using PyMOL software (https://www.pymol.org/), and docking effects were evaluated by the affinity value.

## Data Availability

The datasets used or analyzed during the current study are available from the corresponding author on reasonable request.
